# Popliteal Arteriovenous Fistula: A Rare Complication of Knee Arthroscopy

**DOI:** 10.7759/cureus.81237

**Published:** 2025-03-26

**Authors:** Harminder Sandhu, Sonal Kaushik, Michael Hudson, John Iljas

**Affiliations:** 1 Radiology, Michigan State University, Detroit, USA; 2 General Surgery, Detroit Medical Center Sinai Grace, Detroit, USA; 3 Vascular Surgery, Detroit Medical Center Sinai Grace, Detroit, USA

**Keywords:** arteriovenous fistula, arteriovenous fistula (avf), arthroscopic knee surgery, general and vascular surgery, iatrogenic popliteal fistula, popliteal artery, popliteal pseudoaneurysm, surgical case reports, surgical complication, vascular surgery

## Abstract

Arthroscopic surgery, especially of the knee, is a minimally invasive procedure with low rates of complications. Rarely, this procedure can be associated with traumatic arteriovenous fistula (AVF) formation secondary to popliteal arterial injury. Here, we present the case report of a 33-year-old woman with symptomatic iatrogenic popliteal arteriovenous fistula formation following right knee arthroscopy. The patient has a 10-year history of left knee pain secondary to an unspecified knee injury. Magnetic resonance imaging (MRI) revealed loose bodies within the knee joint and a medial meniscal tear. She underwent left knee arthroscopy with the removal of loose bodies, partial medial meniscectomy, medial femoral chondroplasty, and debridement. Two weeks following arthroscopy, the patient developed new-onset left calf pain, edema, tenderness, and left lower extremity claudication with difficulty ambulating. A physical examination and imaging workup revealed a left popliteal arteriovenous fistula and associated venous pseudoaneurysm. The patient then underwent open surgical takedown of the arteriovenous fistula with patch angioplasty repair of the popliteal artery and primary repair of the popliteal vein and associated pseudoaneurysm. Postoperatively, the patient progressed well with the resolution of calf pain and edema and the return of ambulation to her baseline. Traumatic AVF formation following iatrogenic vascular injury during knee arthroscopy is a rare phenomenon. These lower extremity AVFs can lead to devastating limb-threatening complications, especially with delayed diagnosis and treatment. Despite our patient having a good physical outcome following AVF repair, this complication could have led to permanent disability and limb loss if not identified and addressed in a timely fashion. Physicians should have a high index of suspicion for the possibility of vascular injury after knee arthroscopy, especially if surgery involves the knee's posterior compartment. Preventative measures include avoiding unnecessary trauma, ensuring knee flexion, and avoiding excessive manipulation of the knee during surgery. This case adds to the limited literature on the topic of iatrogenic popliteal AVF formation as a potential complication of orthopedic surgery and discusses the prevention, diagnosis, and repair of vascular injury following knee arthroscopy.

## Introduction

An arteriovenous fistula (AVF) is an abnormal connection between an artery and a vein that shunts blood directly from the arterial to the venous circulation. AVFs may be congenital, also known as arteriovenous malformations, or acquired. Acquired AVFs can be surgically created for the purpose of hemodialysis or can be caused by an insult to vascular structures as a result of trauma and the encroachment of an artery into a vein. The sources of trauma can include blunt, penetrating, or iatrogenic injury. Iatrogenic trauma is a growing cause of AVFs caused by invasive medical procedures such as cardiac catheterizations and percutaneous biopsies [1]. The symptoms vary based on fistula characteristics and location, with the most common findings being a thrill or bruit over the affected area. Patients can also present with warmth of overlying skin, a palpable pulsatile mass, arterial ischemia, venous insufficiency, or high-output heart failure. AVFs are initially diagnosed via duplex ultrasonography, and most acquired lesions are treated with open surgical or endovascular procedures. Arthroscopic surgery, especially of the knee, is considered a relatively safe minimally invasive procedure with low rates of complications; however, there have been reported cases of AVF formation following arthroscopic surgeries of the temporomandibular joint (TMJ) and knee [2-6]. Iatrogenic AVF formation following knee arthroscopic surgery is rare and can be dependent on the type of knee arthroscopic surgery performed [7-13]. In this report, we present a case of traumatic popliteal arteriovenous fistula creation following knee arthroscopy and its surgical management. This article was previously presented as a meeting abstract at the 2nd Annual Michigan State University College of Osteopathic Medicine (MSUCOM) Research Day on April 11, 2024.

## Case presentation

**Figure 1:**
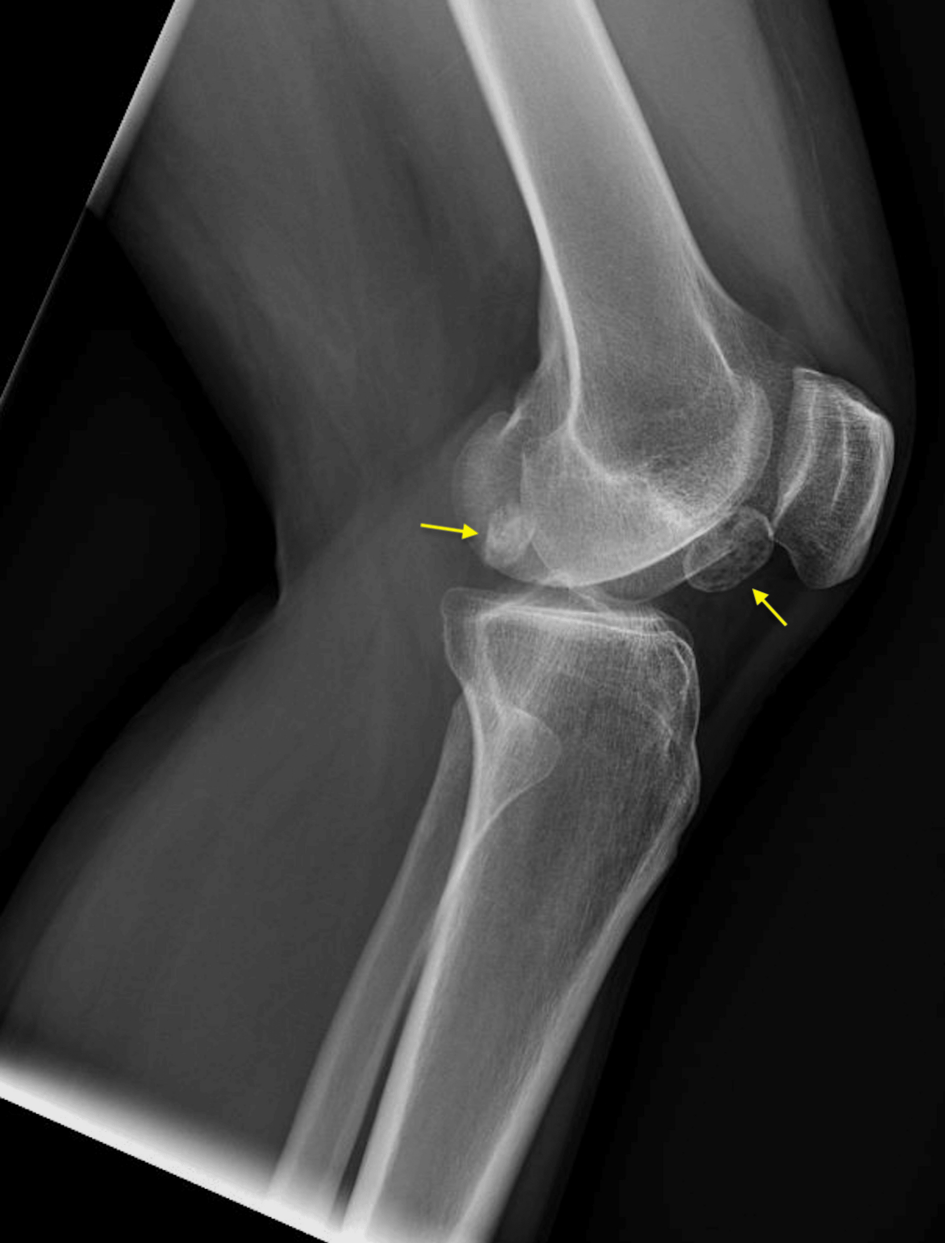
X-ray of the left knee (lateral view) showing multiple intra-articular loose bodies (yellow arrows).

**Figure 2:**
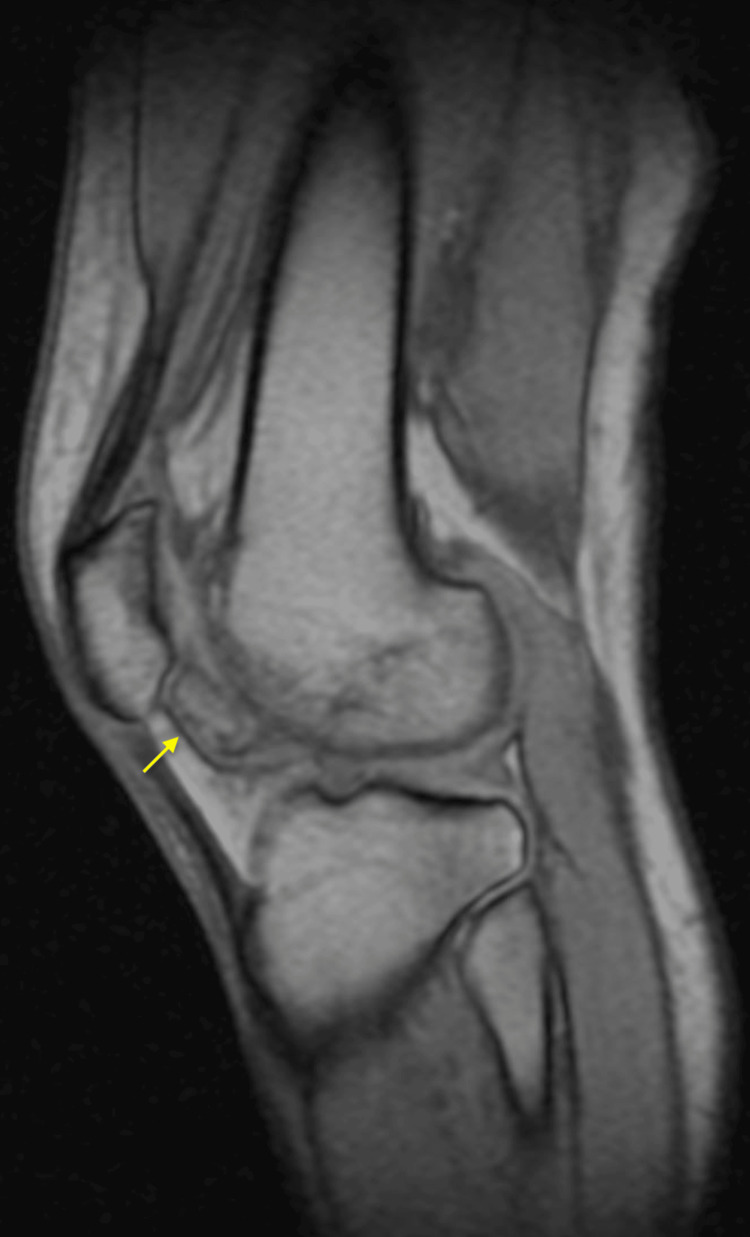
Magnetic resonance imaging of the left knee (sagittal view) showing an intra-articular loose body (yellow arrow).

The patient is a 33-year-old woman with a past medical history of osteochondritis dissecans, chondromalacia, anorexia nervosa, and major depressive disorder who initially presented to the emergency department with a 10-year history of persistent left knee pain limiting her ability to ambulate and exercise safely. A few years ago, the patient had a magnetic resonance imaging (MRI) of the left knee, which showed loose bodies within the joint. At that time, she was offered physical therapy, which she stated did not alleviate her symptoms. Her physical examination was negative for left knee effusion or palpable loose bodies. She had a smooth knee range of motion from zero to 110 degrees, knee stable to varus and valgus stress, no anterior-posterior drawer laxity, and no audible sound with the lateral McMurray's test. However, there was mild pain with the medial McMurray's test. A left knee X-ray was performed, which showed mild medial-lateral joint space narrowing, a small suprapatellar effusion, and multiple loose bodies that appeared to be intra-articular (Figure 1). A follow-up magnetic resonance imaging (MRI) study was conducted, which showed free-edge fraying of the body of the medial meniscus, unicompartmental medial chondromalacia, pes anserine bursitis/synovitis, a partial tear of the posterolateral anterior cruciate ligament (ACL) bundle at its tibial insertion, and three ossified loose bodies (Figure 2).

The patient was offered a left knee arthroscopy by the orthopedic surgery team with the removal of loose bodies and left knee medial meniscus repair versus partial meniscectomy in two months with the goal of the surgery being to reduce the patient's pain and improve her activity level. She was agreeable to operative intervention and underwent a partial medial meniscectomy, chondroplasty, and debridement. Additionally, two loose bodies, one large 6 cm × 3 cm in the medial gutter and another 3 cm × 3 cm in the posterior compartment, were removed. There were no reported complications during the arthroscopic surgery, and the estimated blood loss was 10 cc.

**Figure 3:**
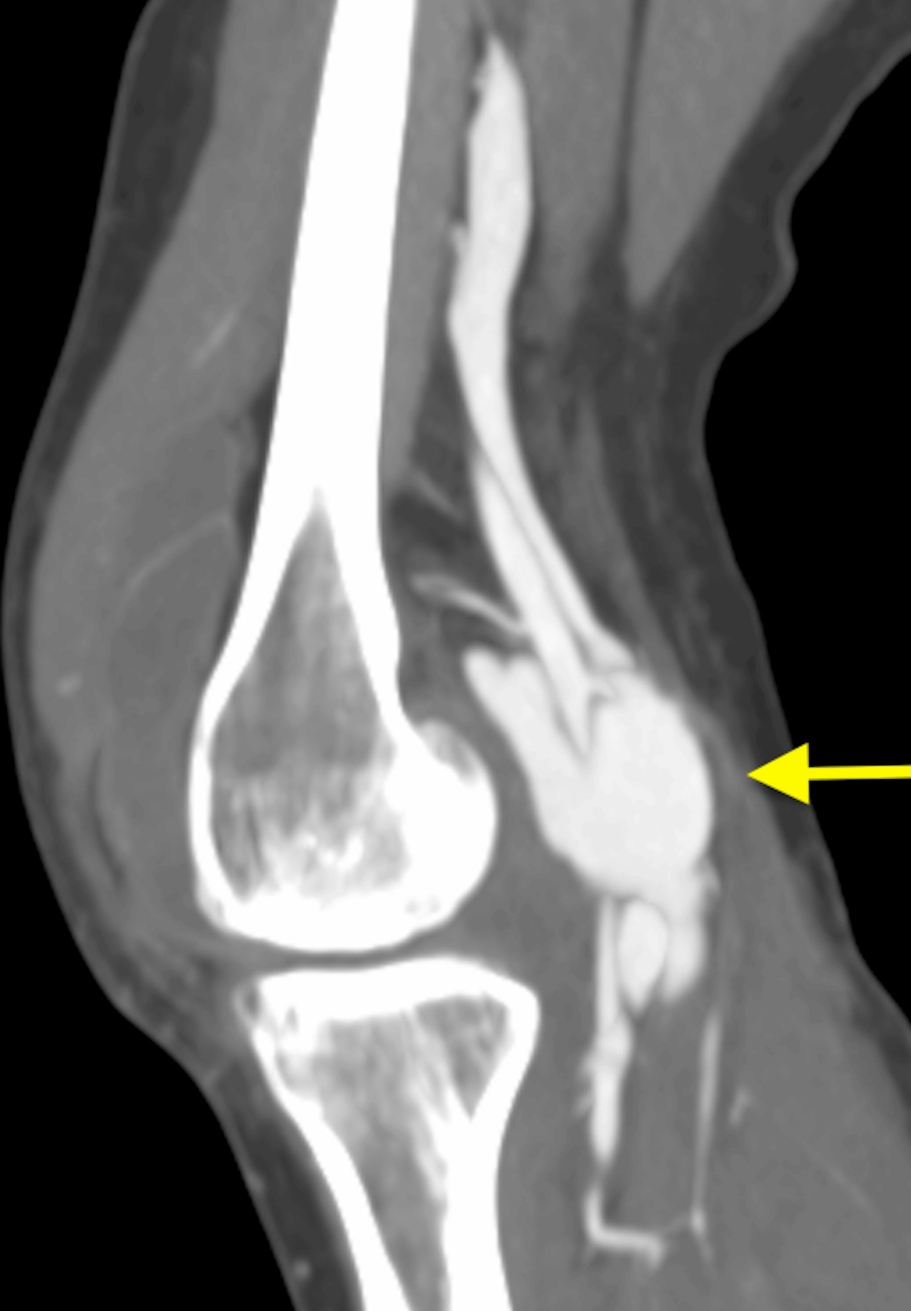
Computed tomography angiogram of the left knee with contrast (sagittal view) showing popliteal vein dilation (yellow arrow).

**Figure 4:**
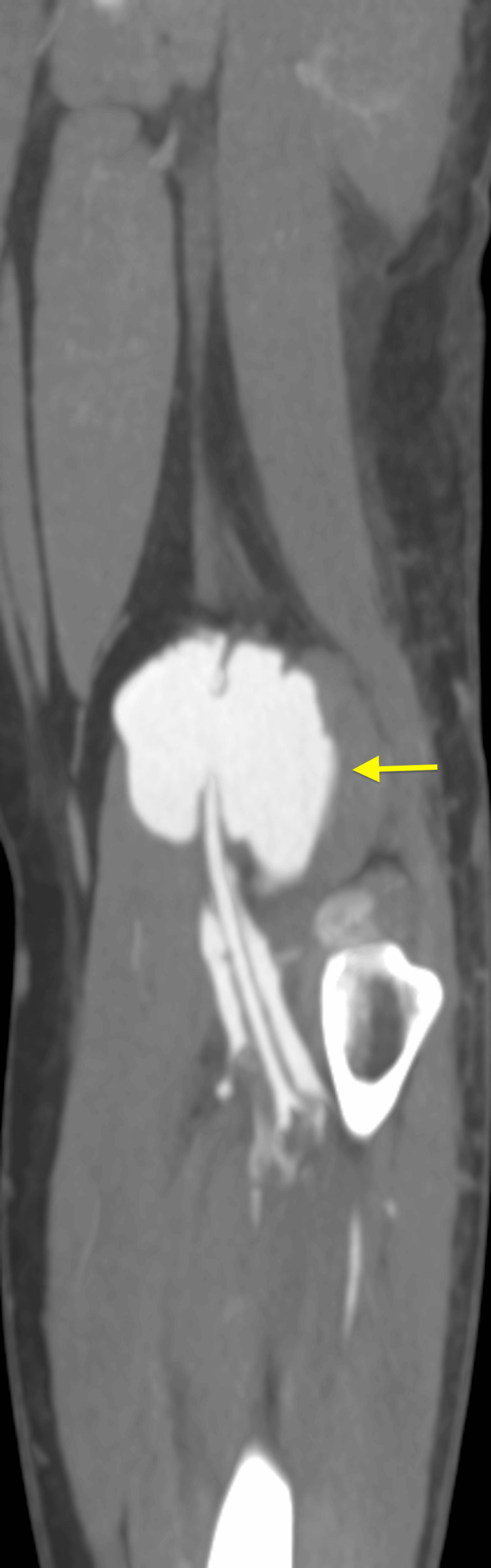
Computed tomography angiogram of the left knee with contrast (coronal view) showing popliteal vein dilation (yellow arrow).

Approximately two weeks following the procedure, the patient returned to an orthopedic clinic for her first postoperative visit. The patient stated initially that her pain had improved; however, three days postoperatively, she began experiencing increased left knee pain (9/10 on the visual analogue scale {VAS}) and swelling with associated constant left calf tenderness, which she described as tight pressure. She was utilizing crutches for ambulation. The physical examination at that time revealed left calf tenderness and edema, no motor or sensory deficits in the distal lower extremity, and an intact left lower extremity pulse. Given the calf edema and pain, the patient underwent a lower extremity vascular duplex to rule out a deep vein thrombosis. No evidence of acute or chronic deep or superficial venous thrombosis was identified within the left lower extremity. However, the ultrasound duplex studies revealed a pseudoaneurysm versus an arteriovenous fistula of the popliteal artery and vein. Subsequently, the patient was sent to the emergency room (ER) where a computed tomography angiogram (CTA) of the left lower extremity with intravenous contrast was conducted that showed a large left suprapatellar joint effusion and an aneurysmal dilation of the left popliteal vein measuring 4.4 cm × 3.0 cm × 6.6 cm (Figures 3, 4). This alongside the duplex ultrasound imaging suggested the presence of a left popliteal venous pseudoaneurysm with concern for possible traumatic AVF.

**Figure 5:**
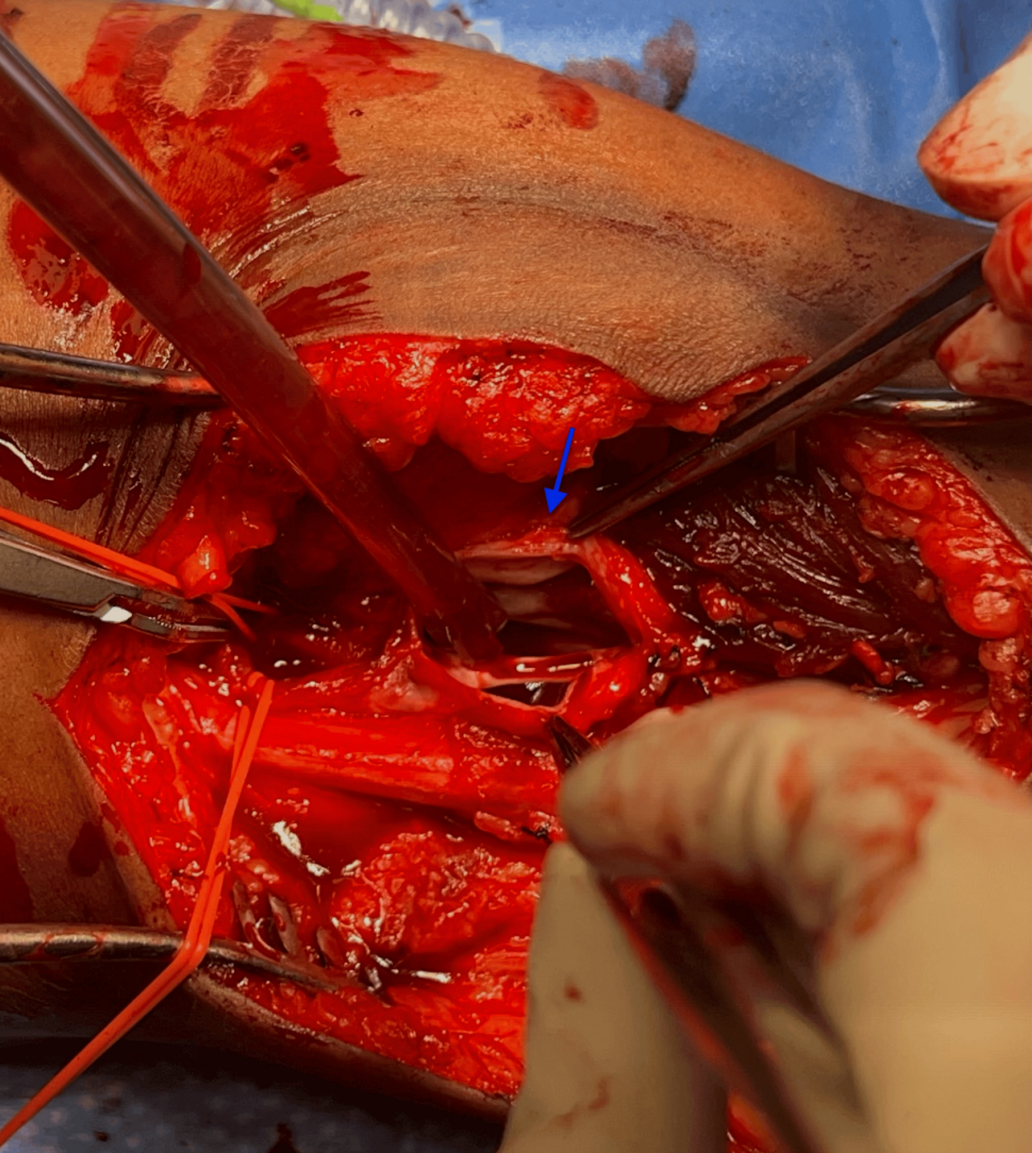
Intraoperative picture of the left knee (posterior view) showing the popliteal artery defect (blue arrow).

Vascular surgery was consulted, and on physical examination, the patient had a palpable thrill in the left popliteal fossa and intact left distal lower extremity pulses with no motor or sensory deficits. The patient was then seen in the vascular surgery clinic for follow-up with continued complaints of pain, edema, and claudication in the left lower extremity; thus, she was offered surgical repair of the traumatic left popliteal AVF. She underwent an open repair in the prone position. A lazy S incision was made starting laterally on the posterior thigh and extending medially on the posterior leg. Intraoperatively, she was noted to have a ruptured popliteal artery, which appeared to have fistulized with the adjacent popliteal vein (Figure 5). Following the takedown of the AVF, the popliteal arterial defect was repaired using a XenoSure (LeMaitre Vascular, Inc., Burlington, MA) bovine pericardium patch angioplasty, while the popliteal vein was repaired primarily. The patient had an estimated blood loss of 200 cc. She was admitted to the hospital postoperatively. While an inpatient, she was ambulating with minimal pain, and her distal lower extremity pulses remained strong and intact. She was discharged home on postoperative day 2 on dual antiplatelet therapy with aspirin and Plavix for three months. The patient was seen in the vascular surgery clinic two weeks postoperatively and with near complete resolution of left lower extremity edema and 0/10 pain on the VAS.

## Discussion

Arthroscopic surgery of the knee is a minimally invasive procedure that is generally safe and widely performed [2-4]. According to a national survey conducted by the Committee on Complications of the Arthroscopy Association of North America, arthroscopic knee surgery had an overall complication rate of 0.56% [4]. Vascular complication rates were approximately 0.0032% with the popliteal artery being most commonly affected, and the complication rate associated with meniscal repair was 2.4% [4]. Although AVF formation following arthroscopic surgery is very rare, there are some cases in the literature discussing AVFs affecting various vessels following knee arthroscopic surgery [7-13] including the superior medial geniculate, inferior medial and lateral geniculate, descending geniculate, recurrent anterior tibial, and, as in this case, the popliteal artery [7,11,12].

The exact mechanism of popliteal artery AVF formation following arthroscopic knee surgery is not well studied, likely due to the rarity of such cases and the subsequent dearth of research investigating their causal mechanisms. However, injury to the popliteal artery by indirect or direct mechanisms, such as surgical instrumentation, the use of tourniquets, knee hyperextension, or hyperflexion, can lead to inflammatory processes involving the vasculature, which in turn lead to the formation of fistulas [13]. In our case, it was likely a combination of factors including direct injury as a result of arthroscopy and possibly excessive knee flexion or extension, which can lead to vessels contacting sharp edges of bones or loose cartilaginous tissue as our patient had [14]. The popliteal artery and its associated neurovascular bundle move furthest posteriorly from the posterior aspect of the knee joint in 90 degrees of flexion; the popliteal artery is located around 3 mm lateral to the posterior cruciate ligament (PCL) in flexion (2.4 mm in extension) and 10 mm posterior to the knee joint in 60-90 degrees of flexion (5 mm in extension) [14-16]. Therefore, conducting the surgery with appropriate knee flexion and avoiding excessive manipulation of the knee can reduce the risk of popliteal artery injury [14]. It is possible in our case that manipulation during the arthroscopy of the posterior compartment of the knee, in close proximity to the PCL in a flexed position, could have led to a popliteal arterial wall injury.

Despite the rarity of popliteal artery injury leading to AVFs, this complication can lead to devastating limb-threatening complications, such as leg amputations and death, especially when treatment is delayed. Traumatic AVFs are most often diagnosed within one week, but some may be identified many weeks or years later [17]. For this reason, along with methods to prevent AVFs, prompt diagnosis and referral to vascular surgery are vital. To accomplish this, physicians should have a high index of suspicion for the possibility of vascular injury after knee arthroscopy, especially if surgery involves the posterior compartment of the knee. In our case, the patient was diagnosed around two weeks post surgery immediately following her first postoperative clinic visit, even though the patient had symptoms for multiple days prior to presenting at the clinic. Patients who have had arthroscopic knee surgery should be made aware of the signs and symptoms of AVFs and other complications for which they should seek out immediate medical attention, since some patients may ignore subtle symptoms. The patient in our case had an increase in swelling in the knee, constant calf tenderness, and progressive difficulty with ambulation. Despite the patient in our case having a good physical outcome following the AVF repair, this complication may have had a significant impact if not recognized in a timely fashion.

## Conclusions

In conclusion, AVFs are a rare complication after knee arthroscopy that can lead to significant morbidity and limb-threatening complications. Early identification and management are key to avoiding these serious complications with special attention toward patients who have had posterior compartment surgery. The most important ways to prevent AVFs following knee arthroscopy would include avoiding unnecessary trauma or procedures, ensuring knee flexion, and avoiding excessive manipulation of the knee during the surgery. Importantly, patients should be educated on the signs and symptoms of AVFs and other postsurgical complications regardless of their rarity, to ensure patients are educated to seek medical attention when warranted and prevent any potential long-term adverse outcomes.
